# Chemistry in Bioinformatics

**DOI:** 10.1186/1471-2105-6-141

**Published:** 2005-06-07

**Authors:** Peter Murray-Rust, John BO Mitchell, Henry S Rzepa

**Affiliations:** 1 Unilever Centre for Molecular Science Informatics, Department of Chemistry, University of Cambridge, Lensfield Road, Cambridge. CB2 1EW, UK.; 2 Department of Chemistry, Imperial College London, SW7 2AY, UK.

## Abstract

Chemical information is now seen as critical for most areas of life sciences. But unlike Bioinformatics, where data is openly available and freely re-usable, most chemical information is closed and cannot be re-distributed without permission. This has led to a failure to adopt modern informatics and software techniques and therefore paucity of chemistry in bioinformatics. New technology, however, offers the hope of making chemical data (compounds and properties) free during the authoring process. We argue that the technology is already available; we require a collective agreement to enhance publication protocols.

## Introduction

In "Representation and Use of Chemistry in the Global Electronic Age” [1] we showed that new technology can provide great increases in the quantity and quality of aggregated chemical information published in the primary literature. We also argued the benefits of Open Access and Open Data. The current invited overview and a parallel technical article extends the same methodology to chemistry in bioinformatics to remove the loss and corruption of data that occurs in current publishing. We are pleased that this article is an Open Access publication, and we expect that bioinformatics, with its culture of Open Data, is more likely than mainstream chemistry to adopt new approaches. The benefits of open access  include higher quality, greater availability, and development of the Biochemical Semantic Web where robots mine text and data as a basis for knowledge-driven science. We argue that funders, institutions, authors, editors, publishers and readers will all benefit.

Biosciences now require large amounts of detailed chemical information, examples of which include the  occurrence and role of small-molecules in biological processes; the mechanism of biochemical reactions and interactions; the structure and properties of biomolecules; reagents, protocols and classificatory tools for performing bioscience; chemistry in the ecosphere. Such information is only available in a dispersed manner in the primary literature and current mechanisms for its collection and dissemination do not meet the needs of bioscience. However if there is a communal will, modern chemical informatics technology can provide what is required. Several excellent models for the capture of macromolecular sequence and structure data (e.g. the protein data bank) inform our architecture.

Data in published articles can include reference to chemical compounds (often in free text), details of their synthesis (in vivo and in vitro), proof of their structure (spectra and analytical data), methods and reagents in bioscience protocols, the physical and biological properties and reaction of compounds both in enzymes and enzyme-free systems. With the tools that we and others have developed, this information can now be automatically captured with high precision from primary publications, especially if structured authoring tools are widely used.

Unlike bioinformaticists, who routinely  apply data- and text-mining tools in their research, conventional chemists appear culturally more suspicious of robotic data extraction and continue to rely on manually curated secondary publications whose philosophy has barely altered over 120 years. Such sources are necessarily incomplete in time, coverage and coverage of information types. For example for 99+% of all newly synthesised compounds the papers report that Infra-red spectra have been recorded, but only a tiny fraction of this is available in electronic form. We argue that even modest improvement in such a data capture rate would make an enormous difference. Moreover the data would be of consistently higher quality than manually re-keyed data. The main challenge is a cultural one. Thus biosciences and crystallography have communally convinced authors and publishers of the value of author-based deposition of data, later aggregated in communally accessible databanks. This largely does not currently happen in chemistry, where information is manually extracted from the primarily literature, jealously guarded and sold back to the community. Mechanisms such as "supporting or supplemental data deposition" are not widely used, and when they are, little care is given to enabling its re-use. One of the major secondary publishers has recently criticised the bioscience community for aggregating chemical data. 

"*It [PubChem] would not only injure us significantly, it would put information for free in the hands of world scientists and do it all with taxpayer money," Massie [CEO, Chemical Abstract Service] said. "For me to wake up one morning and find I have to compete with my own government is extraordinary.”*[2]

The attitude in chemistry to modern informatics (XML, ontologies, RDF, text-mining, metadata, etc.) [3] is largely apathetic, with some data- or software-centric organisations actively opposing interoperability for commercial reasons. This problem extends to mainstream chemical software, where there are no Open standards and where algorithms are closed and obscure. We have argued that the large data aggregators produce vendor-centric access systems to meet their needs rather than the community's. Another problem is that access is often only allowable on a per-item basis rather than to the data collection as a whole. This monopolistic "thought control" in chemistry stifles innovation in data-led science. However the Opens (Data, Source, Access, Standards) are changing the practice of scientific informatics and chemistry is starting to be affected.

We therefore look to bioscience to take a lead in helping realise the following vision. We now believe that there are already enough Open tools and Open resources which can make the vision attractive and cost-effective.

### A model for automatic capture of chemical information

Much chemical data is largely context-free in that it can be understood and recreated independently of the location or motivation. The primary data model, inspired by Konrad Beilstein in the 19th century, has three components: compound, properties and citations. A pure compound is described by an immutable structural formula and has precisely reproducible properties. Current thinking asserts that the biological action of a compound is, in principle, reproducible and predictable if the system is carefully enough replicated and the components understood. This is the central dogma of the chemically-based pharmaceutical industry and the chemical information industry on which it relies.

Chemistry has a tradition of ensuring quality through reporting properties and analysis, so every new compound (and many re-synthesised ones) must have published measurements of properties to justify their identity and purity. These facts are available, in text form, in the primary literature in which over a million new compounds are published annually. Because structure predicts properties, and because drug discovery is so difficult, the pharmaceutical industry tests many compounds for biological activity. The data in these public publications is a major feedstock for the chemical information industry.

The chemical bioscientist has almost all of the required information available in electronic form on their benchtop already! It could be deposited for the scientific community with virtually no human intervention. We believe that, with the help of forward looking publishers, a working protocol can be set up in bioscience, which will then inspire (or terrify) mainstream chemical informatics. Note that much of the information captured is additional to that which the current abstracters collect.

We argue that the key components to automatically capture chemical information are already in place (and are discussed in more detail in an accompanying technical article). We envisage the chemistry which can be captured using such mechanisms includes (a) Chemical entities and names. Many compounds have no explicit structures and are mentioned only by name or identifiers. Where these relate to specific compounds it is valuable to link them to a precise identification, such as PubChem. (b) Molecular structure, expressed as a compositional formula (e.g. CHaOH for methanol) and a graphical structural formula ("2D diagram" or connection table). (c) Spectra and physical properties. Much such information is already in digital form when produced by instruments (whose manufacturers are starting to create Open approaches [4], but is largely destroyed by conventional publishing processes If a community-wide digital template for the submission of this information were available and encouraged by publishers it would be welcomed by many, would eliminate errors introduced by transcription, and enable machine-reviewing of data leading to a higher standard of published data.

The basis of our model involves conversion of experimental data to XML and its merger with the conventional text (giving a “datument” [5]). The author uses a authoring tool which can manage structured XML documents and provide normal textual support (spellchecks, etc.). The resulting datument contains fine-grained markup of facts (molecules, measurements, properties, chemical names) and can automatically be used to create derivatives such as the "full-text" or the "supplemental data". The complete datument, if Open, or the "data" if not is then reposited for further harvesting. All compound/property data is available for datamining and computational re-use (e.g. for further in silico prediction.

### Realising the vision

#### Data repositing and maintenance

The current dissemination of data through publishers is largely unsatisfactory.   Thus although many publishers allow the deposition of factual "supplementary data”,  our experience with most is that it is an unwelcome chore, poorly resourced and maintained. Moreover although reviewers are often do what they can to validate data, publishers themselves do not. We believe that many publishers would welcome a model where they were no longer involved in data repositing. A few publishers such as the International Union of Crystallography are more committed to the curation of data; others in the biosciences see the value of semantically enhanced data.The crystallographic experience has shown that expert computer programs can act as powerful reviewers complementing the human; automatic curation enhances, rather than lowers, data quality.

Our model is based on the availability of repositories, primarily Institutional, that accept data as well as full text. Already some academic institutions and an increasing number of funders mandate that research output should be reposited and there are national initiatives to develop the infrastructure. The storage for XML-ised chemical data is modest (less than 1 mbyte per publication) and we have shown that large numbers of molecules can be deposited in our own institutional repository [6] and recovered by undirected search engines such as Google [7]. Chemical data has required no semantic maintenance (e.g. through changes in meaning or use) over many decades and we see this continuing, so that the maintenance costs are those general to any repository.

Components in a repository have a unique handle with which,  in principle, a Digital object or other identifier (DOI) [8] can be associated so that data can be cloned for access and preservation. The handle or DOI would be published in the "full text" and would bind the data to it more effectively that at present and hopefully indefinitely.

#### Metadata

Through the InChI (International Chemical Identifier)[8] and a controlled vocabulary of chemical properties, generic search engines can achieve a very high degree of recall. This means that discovery and aggregation can be built on maintenance-free generic technology and can be made completely automatic, Conventionalists would argue of course that human curation is essential for re-usable chemical data. In a similar vein to much bioinformatic, we argue that robots can discover patterns in data, compounds and authors which are at least as powerful as many current abstracting services. Where human evaluation is critical (e.g. in human medicine, patents, etc.) then the robots will provide the primary resources on which a judgement can be based.

#### Rights

We assume that most bioscience authors and publishers will agree that whether or not a paper is Open Access the facts (and thereby all "supplemental data") therein are not copyrightable. XML resolves differences of interpretation in that XML markup can be regarded as identifying factual information and this would be consistent with its re-use under (say) the Budapest Open Access Initiative. In this way all published chemical data can be made immediately, completely and clearly available for indefinite scientific re-use.

#### Potential

Because the chemical information is structured we now have a biocheminformatics "cycle" where, for the first time, large scale robotic data analysis can take place. The data in the research (laboratory, in silico, or both) are published in a lossless manner. Molecules and their properties have unique identifiers as described above and can be integrated into mainstream bioinformatics in the same manner as collections such as PubChem, the macromolecular stucture database (MSD at EBI), the Kyoto Encyclopedia of Genes and Genomes (KEGG) etc.  They will bring the added value of consistently captured property data and spectra. We also expect that many  in silico properties will then be systematically added.

#### Author and publisher compliance

The introduction of structured authoring tools (e.g. Publicon) [9] will help this process considerably. Templates can be created for the chemical components described above and where the information exists in XML (connection tables, spectra, properties) it should be as easy as for committed authors as using a semantically void tool (e.g. Word). Where information needs to be converted from legacy formats, an increasing number of open Web Services,  which publishers (and authors) may clone and customise are becoming avilable. We expect authors to have a greater incentive (even if only through mandation) to reposit data and to disseminate research findings. This also raises the vision of changing the "citation economy" (which values market perception) to a "reuse economy" where a the data in a paper are valued by how often they are re-used.

## References

[B1] Murray-Rust P, Rzepa HS, Tyrell SM, Zhang Y (2004). Org Biomol Chem.

[B2] Government-funded Free Information for Chemists 'Unfair' Competition for Private Monopolies. http://www.okfn.org/drn/node/56.

[B3] World Wide Web consortium. http://www.w3c.org/.

[B4] Analytical Information Markup Language. http://animl.sourceforge.net/.

[B5] Murray-Rust P, Rzepa HS (2004). The Next Big Thing: From Hypermedia to Datuments. J Digital Inf.

[B6] University decision to offer free online access to all research. http://www.jisc.ac.uk/index.cfm?name=free_access_to_university_research_news171204.

[B7] Coles SJ, Day NE, Murray-Rust P, Rzepa HS, Zhang Y (2005). Enhancement of the Chemical Semantic Web through the use of  InChI Identifiers. Org Biomol Chem.

[B8] Murray-Rust P, Rzepa HS, Stein S (2004). The InChI as an LSID for molecules in lifescience. W3C Workshop on Semantic Web for Life Sciences, 27-28 October 2004, Cambridge,  Massachusetts USA.

[B9] BioMed Central and *Publicon*. http://www.biomedcentral.com/info/ifora/publicon/.

